# Endophytic Fungal Metabolites as Modulators of Key Signaling Pathways in Chronic Diseases and Aging

**DOI:** 10.3390/antibiotics15080799

**Published:** 2026-08-18

**Authors:** Asiya Nazir, Prathap Bava, Arif Hussain, Touseef Amna, Mohammad Chand Jamali, Afsheen Raza, Jayanthi Barasarathi

**Affiliations:** 1 College of Health Sciences, Abu Dhabi University, Abu Dhabi P.O. Box 59911, United Arab Emirates; 2 Division of Drug Discovery & Development Research, Cancer Research Institute, Office of Research and Sponsored Programs, Abu Dhabi University, Abu Dhabi P.O. Box 59911, United Arab Emirates; 3 Manipal Institute of Health & Life Sciences (MIHLS), Manipal Academy of Higher Education, Dubai P.O. Box 345050, United Arab Emirates; prathap.bava@manipaldubai.com (P.B.); arifhussain@manipaldubai.com (A.H.); 4 Department of Biology, Faculty of Science, Al-Baha University, Al-Baha 65779, Saudi Arabia; touseefamna@gmail.com; 5 College of Medical and Health Sciences, Liwa University, Abu Dhabi P.O. Box 41009, United Arab Emirates; mohammad.jamali@lu.ac.ae; 6 Faculty of Health & Life Sciences (FHLS), INTI International University, Nilai 71800, Negeri Sembilan, Malaysia; jayanthi.baras@newinti.edu.my

**Keywords:** AGE–RAGE, aging and health span, anticancer effects, antidiabetic regulation, chronic disease pathways, endophytic fungi, glycation, immune modulation, inflammation, microbiome-linked mechanisms, neuroprotection, oxidative stress

## Abstract

Chronic diseases and aging-related disorders are driven by interconnected mechanisms, including oxidative stress, low-grade inflammation, metabolic dysregulation, and glycation. Targeting these overlapping pathways remains a major challenge for conventional single-target therapeutics. In this context, endophytic fungi have emerged as a promising source of bioactive metabolites with multi-target pharmacological potential. This review provides a mechanistic overview of endophyte-derived metabolites, including alkaloids, terpenoids, polyketides, and phenolic compounds, with a focus on their ability to modulate key signaling pathways such as NF-κB, Nrf2, PI3K/Akt, AMPK, and the AGE–RAGE axis. Evidence from experimental studies suggests that these metabolites exhibit anticancer, anti-inflammatory, antioxidant, and metabolic regulatory effects through coordinated modulation of cellular signaling networks. Several endophyte-derived metabolites also possess antimicrobial activity against bacterial and fungal pathogens and may represent a promising source of novel anti-infective agents. Their ability to modulate host immune responses and microbial-associated signaling pathways further highlights their relevance for antimicrobial discovery and microbiome-based therapeutic strategies. Particular attention is given to pathway-level convergence in chronic diseases, including cancer, diabetes, and inflammation-associated disorders, as well as their relevance to aging and health span. The pharmacological potential of these compounds is discussed alongside key limitations, including issues related to bioavailability, reproducibility, and translation into clinical applications. Overall, endophytic fungal metabolites represent a structurally diverse and mechanistically rich resource for the development of multi-target therapeutic strategies. Future integration of metabolomics, genome mining, and advanced disease models will be essential to bridge the gap between experimental findings and clinical application.

## 1. Introduction

Chronic illnesses continue to increase worldwide, largely driven by the growing aging population. These conditions contribute significantly to global disability and mortality and place a heavy burden on healthcare systems, while also affecting healthy aging. In 2019, about 74% of global deaths were attributed to chronic conditions, compared to 67% in 2010 [1]. Chronic diseases, including cardiovascular disorders, diabetes, neurodegenerative conditions, and cancer, represent a major global health challenge that requires approaches beyond individual patient-level interventions [2].

These diseases are difficult to control because they do not act through a single pathway. Instead, they involve interconnected processes such as inflammation, oxidative stress, metabolic dysregulation, and genetic susceptibility [3]. Naturally derived bioactive compounds have contributed in many ways to targeting these pathways, but their structural complexity and variability can limit reproducibility and large-scale application [4,5].

Because of these limitations, attention has gradually shifted toward endophytes. Endophytes are a diverse group of microorganisms, including fungi and bacteria that live within plant tissues without causing visible harm occupying a unique and often overlooked biological niche [6]. These organisms are influenced by their host plants and are known to produce a wide range of secondary metabolites with pharmacological activities, including anticancer, antiviral, antibacterial, and antifungal effects [7,8,9]. They represent a rich source of structurally diverse compounds such as flavonoids, alkaloids, steroids, polyphenols, terpenoids, and tannins.

Endophytes are also increasingly recognized as part of the extended microbiome. This concept highlights how host-associated microbes influence not only their immediate host but also broader biological systems, including plants, animals, and the environment [10]. This perspective aligns with the One Health framework, which emphasizes the interconnectedness of human, animal, and ecosystem health [11]. Endophyte metabolites not only support plant function but also contribute to ecological balance and, in some cases, produce compounds relevant to human health [12].

At the same time, the growing interest in microbiome-based therapeutics has highlighted how microbial metabolites can influence inflammation, metabolism, and immune pathways linked to chronic diseases [13]. In this context, endophyte-derived metabolites are increasingly being explored alongside microbiome-derived compounds, as both target overlapping pathways associated with aging and long-term disease [14].

Endophyte-derived compounds are now recognized as a promising and relatively underexplored source of microbial therapeutics, including anticancer, antimicrobial, antioxidant, and antiviral agents [15]. These metabolites are being studied for their roles in immunomodulation, metabolic regulation, and neuroprotection, often showing functional similarities to metabolites produced by gut and environmental microbes. Endophytic fungi, in particular, produce a range of small molecules such as peptides, quinones, and alkaloids that can influence oxidative stress, inflammation, mitochondrial function, and metabolic signaling linked to chronic diseases and aging [16,17].

Previous reviews have primarily focused on the chemical diversity, biological activities, and biotechnological potential of endophytic fungi. In contrast, this review adopts a pathway-centered perspective by examining how endophyte-derived metabolites and metabolite-enriched extracts modulate interconnected signaling networks involved in chronic diseases and aging, including NF-κB, Nrf2, PI3K/Akt, AMPK, MAPK, and AGE–RAGE pathways. Emphasis is placed on distinguishing evidence derived from crude extracts, identified metabolites, and purified compounds to provide a more balanced interpretation of the current literature. The review further integrates these molecular mechanisms with key hallmarks of aging, including oxidative stress, chronic inflammation, mitochondrial dysfunction, impaired autophagy, proteostasis imbalance, and cellular senescence, while discussing their implications for multi-target therapeutics and healthy aging. In addition, the translational potential, antimicrobial activity, microbiome-associated mechanisms, and emerging technologies, including metabolomics, genome mining, and artificial intelligence-assisted discovery, are considered within a One Health framework linking plant–microbe interactions to human health. While endophyte-derived metabolites are often compared with gut microbiome metabolites because they influence overlapping signaling pathways, this comparison is intended as a conceptual and mechanistic analogy rather than a direct quantitative equivalence, as systematic comparisons of exposure, pharmacokinetics, and delivery remain limited.

## 2. Endophytes and Their Bioactive Metabolites

### 2.1. Isolation and Identification

Epiphytic contaminants can easily overgrow internal fungi and distort downstream analyses of endophytes, which makes it necessary to carefully remove them in isolation of endophytic fungi [18,19]. Surface sterilization chemical disinfectants such as ethanol and sodium hypochlorite, and it is important to keep the balance between concentration and exposure time.

Studies reveal that the concentrations of NaOCl fall between 2% and 10%, depending on tissue type and species-specific traits [20,21]. Even small adjustments of their concentration or in timing can influence the survival of type of endophytes, contributing to inconsistent recovery across studies [22]. Accordingly, protocols must be selected with attention to the physical characteristics of the plant tissue and the goals of the study.

After sterilization, internal fragments of the plant are carefully transferred onto nutrient media. The selection of culture medium strongly influences that the selection of culture medium strongly shapes the diversity of recovered isolates. The most widely used medium is Potato Dextrose Agar (PDA) because it supports a broad spectrum of fungal taxa [23]. To expand diversity, researchers often incorporate additional media such as Malt Extract Agar [24,25], MS-based agar enriched with sucrose [26], Sabouraud Agar [27], or nutrient-poor formulations like water agar that help retrieve slower-growing species [28]. Studies also showed that supplementing the media with small quantities of host plant tissue supports host-associated fungi [29]. A slightly acidic pH of 5.8–6.0 is generally optimal for most isolates [24,30], and antibiotics are commonly added to suppress bacterial endophytes [31].

**Figure 1:**
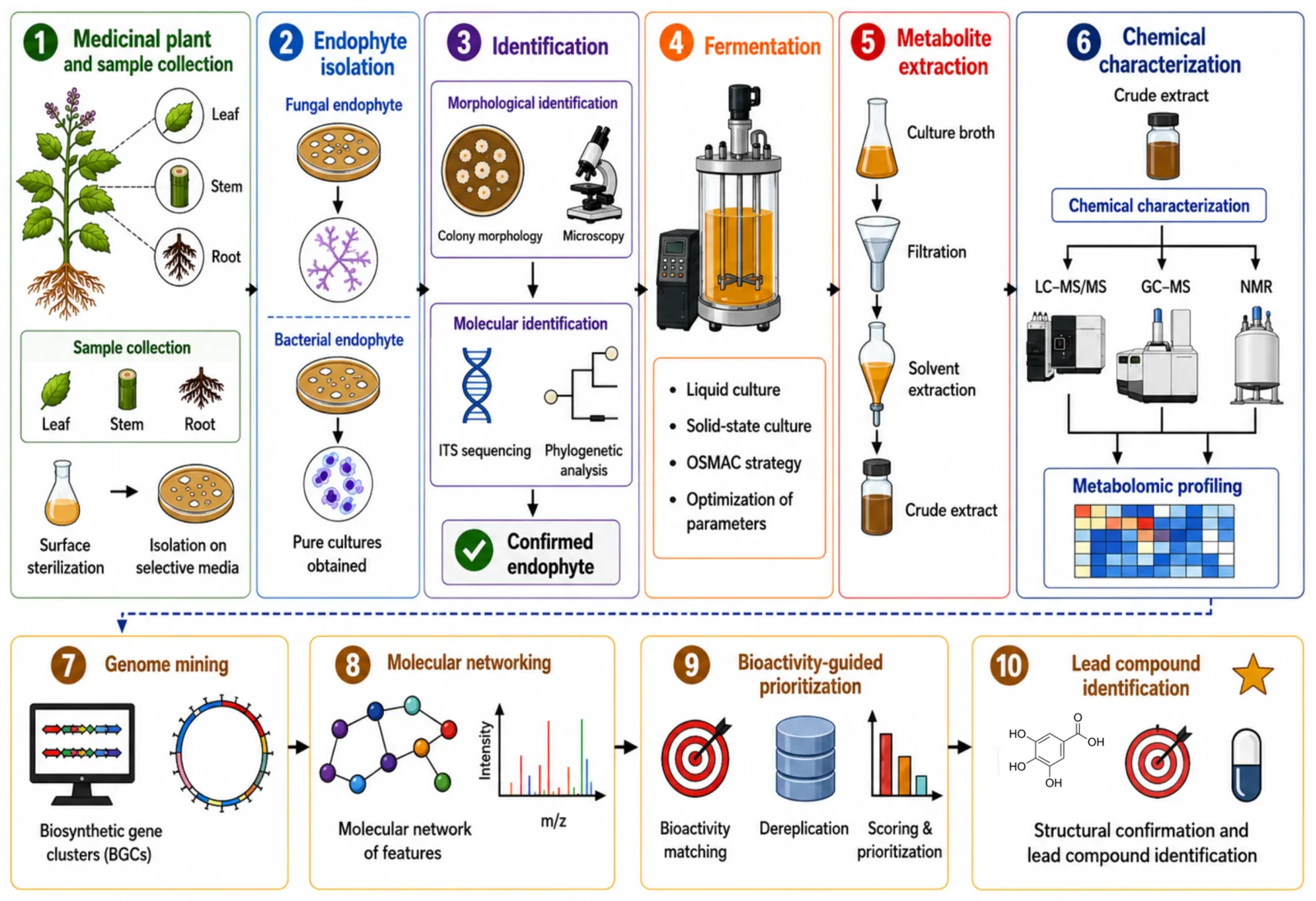
Schematic of the Endophyte-Driven Drug Discovery Pipeline. The figure illustrates the steps from host plant collection and endophyte isolation to metabolite extraction, metabolomic analysis and bioactivity screening, and bioassay screening. (Created in BioRender. Nazir, A. (2026) https://BioRender.com/wlpugxm).

For metabolite studies, purified isolates are often subcultured in liquid media and incubated for 7–14 days at 25–28 °C under shaking conditions, which reliably promotes secondary metabolite production [20,32,33]. Modern workflows (Figure 1) increasingly rely on LC–MS/MS molecular networking to map structurally related metabolite clusters before purification begins [34].

### 2.2. Chemical Diversity of Endophyte Metabolites

Phenolic compounds, including flavonoids, represent one of the most widely reported metabolite classes from endophytic fungi [35]. Many endophytes appear capable of synthesizing phenolic compounds that are structurally similar to those found in their host plants, suggesting that fungal phenolic biosynthesis may be more extensive than previously recognized [36]. Because these compounds are closely associated with antioxidant and anti-inflammatory functions, their presence in endophytes is particularly relevant to chronic diseases and aging biology [37]. Representative endophyte-derived phenolics, flavonoids, and their pathway-level activities are summarized in Table 1. Flavonoid-like metabolites produced by endophytes have also attracted attention for their ability to influence oxidative stress, lipid peroxidation, and inflammatory signaling pathways linked to non-communicable diseases and aging [38]. Endophytes from genera such as *Aspergillus niger*, *Emericella* sp., and *Chaetomium globosum* have been reported to produce phenolic acids, coumarins, benzophenones, and flavonoid-like compounds, with GC–MS analyses identifying metabolites including squalene, hexadecenoic acid, 1-phenyl-2-propanone, and 9,12-octadecadienoic acid within fungal extracts [39,40]. However, several of these observations are still based on extract-level analyses, and definitive attribution to specific fungal metabolites remains under investigation.

Several phenolic acids produced by endophytic fungi have demonstrated anti-AGE activity, which may hold particular significance given the role of glycation in metabolic and hepatic disorders. Protocatechuic acid [41], gallic, rosmarinic, and coumaric acids [42], along with caffeic [43], ferulic [44], and chlorogenic acids [45], are among the compounds commonly detected in endophyte extracts. Flavonoids are also frequently reported, including apigenin, vitexin, isovitexin [46], kaempferol derivatives [47], luteolin [42], quercetin glycosides [48], genistein [43], and rutin-related molecules [49], many of which exhibit comparable anti-AGE potential. Some of these metabolites have additionally shown free radical scavenging activity or modulation of Nrf2-associated antioxidant responses, while others appear to exert antibacterial or anticancer effects through pathways linked to mitochondrial stress and apoptosis [50].

Collectively, these findings suggest that endophytes do more than replicate plant chemistry; they generate metabolites with therapeutic relevance spanning anticancer, anti-inflammatory, antidiabetic, antioxidant, hepatoprotective, neuroprotective, antimalarial, and antiviral activities [51]. Although several mechanisms remain incompletely understood and biosynthetic pathways in fungi are still being explored, emerging genomic and metabolomic studies indicate that fungi may utilize hybrid PKS-shikimate or mixed biosynthetic routes to generate flavonoid-like scaffolds under specific culture conditions. The repeated convergence of these metabolites on pathways associated with oxidative stress, inflammation, mitochondrial stability, and chronic disease progression suggests that their chemical diversity is unlikely to be incidental and warrants deeper investigation as part of microbiome-derived therapeutic discovery.

**Table 1:** Major Chemical Classes of Bioactive Metabolites Produced by Endophytic Fungi and Their Molecular Targets and Therapeutic Relevance.

Metabolite Class	Representative Compound(s)	Representative Endophytic Fungal Source	Primary Molecular Target(s)	Major Biological Activity	Disease Relevance	Reference
Alkaloids	Sanguinarine, Chaetominine	*Fusarium* spp., *Chaetomium* spp.	NF-κB, PI3K/Akt, caspase signaling	Anticancer, anti-inflammatory, pro-apoptotic	Cancer, chronic inflammation	[52,53]
Terpenoids	Libertellenone M	*Phomopsis* spp., *Penicillium* spp.	Nrf2, MAPK	Antioxidant, anti-inflammatory	Oxidative stress, aging	[54,55]
Polyketides	Anthraquinones	*Aspergillus* spp., *Penicillium* spp.	ROS signaling, mitochondrial pathways	Antioxidant, antimicrobial	Chronic inflammation, microbial infections	[53,56]
Phenolics & Flavonoids	Protocatechuic acid, Quercetin derivatives	*Aspergillus* spp., *Chaetomium* spp.	AGE–RAGE, Nrf2	Antioxidant, antiglycation	Diabetes, healthy aging	[50,57]
Peptide-like compounds	Diketopiperazines	*Trichoderma* spp., *Penicillium* spp.	Cytokine signaling, NF-κB	Immunomodulatory, antimicrobial	Immune disorders, inflammatory diseases	[55,58]

## 3. Endophyte Metabolites in Chronic Disease Management

Rochín-Hernández [59] reported that many endophyte-derived metabolites appear to link pathways that lie at the heart of non-communicable diseases, although the strength of these interactions can vary from one compound to another [59]. Studies also show some metabolites reduce protein glycation or interrupt AGE–RAGE signaling, a biochemical thread that links quietly but persistently to oxidative stress, mitochondrial injury, endothelial dysfunction, and several forms of chronic inflammation [14]. Others influence regulators such as NF-κB, PI3K/Akt, AMPK, and MAPKs mechanisms reported across studies analyzing fungal phenolics, terpenoids, and alkaloids, even though their exact contributions are not always straightforward to map [60,61]. Some of these effects overlap at the pathway level with those attributed to gut microbial metabolites, particularly phenolic acids and indole derivatives known to modulate redox balance, immune tone, and metabolic signaling in host tissues [62,63]. While the overlap is conceptual rather than quantitative and the supporting evidence is still emerging, it suggests that endophyte metabolites may occupy a complementary similar therapeutic space within microbiome-focused strategies, contributing molecules that influence the same circuits shaping cancer, metabolic disorders, and chronic inflammatory diseases.

### 3.1. Anticancer Activities

Endophytic fungi have gained considerable attention as a rich source of structurally diverse bioactive metabolites with promising anticancer potential. Several studies have reported the detection of compounds such as paclitaxel, podophyllotoxin, camptothecin, vinblastine, hypericin, and diosgenin, or structurally related analogues, in endophytic fungi associated with host plants including *Thielavia subthermophila*, *Seimatoantlerium nepalense*, *Fusarium redolens*, *Rhizopus oryzae*, *Chaetomella raphigera*, and *Aspergillus fumigatus*. However, for several plant-associated metabolites, independent fungal biosynthesis remains under investigation, and definitive evidence is still lacking in some cases [53,64,65]. Collectively, these studies suggest that endophyte-derived metabolites may interfere with cancer development through a combination of cytotoxic, antiproliferative, and signaling-modulatory effects. However, the supporting evidence ranges from studies using crude fungal extracts to investigations of purified metabolites, and the underlying molecular mechanisms have not been uniformly validated across all compounds. Purified endophyte-derived metabolites have been reported to induce apoptosis through multiple mechanisms, including modulation of Bcl-2 family proteins, activation of caspase cascades, mitochondrial membrane depolarization, cytochrome *c* release, and alterations in the Bax/Bcl-2 ratio [52,66]. In addition, some studies suggest involvement of extrinsic death receptor-mediated apoptosis, although the evidence varies depending on the metabolite and experimental model [67].

Mechanistic studies indicate that purified endophyte-derived metabolites can modulate multiple signaling pathways involved in cancer progression. Several compounds have been reported to induce apoptosis by regulating the balance between pro- and anti-apoptotic proteins, activating caspase cascades, promoting mitochondrial membrane depolarization, facilitating cytochrome c release, and altering the Bax/Bcl-2 ratio, thereby triggering intrinsic apoptotic pathways [52,66]. Some metabolites have also been associated with activation of extrinsic death receptor-mediated apoptosis, although the supporting evidence varies depending on the compound and experimental model [67]. In addition to apoptosis, taxol-like molecules, podophyllotoxin analogues, and quinazolinone derivatives have been reported to disrupt microtubule dynamics, resulting in G2/M cell-cycle arrest in several cancer models [52]. Other purified metabolites have demonstrated modulation of PI3K/Akt/mTOR and MAPK signaling pathways and suppression of angiogenesis- and oxidative stress-associated mediators, thereby influencing tumour cell proliferation, invasion, and metastatic potential [68]. These biological activities are distributed across multiple structural classes, including alkaloids, terpenoids, polyketides, and peptide-derived metabolites [69]. Collectively, the available evidence suggests that endophyte-derived metabolites exert anticancer effects through coordinated modulation of multiple cellular pathways rather than a single molecular target, although further mechanistic validation and in vivo studies are required for many reported compounds [70].

### 3.2. Antidiabetic and Metabolic Regulation

Studies also demonstrated that endophytic fungi yield metabolites that seem to influence glucose regulation through several complementary routes. Their metabolic effects appear surprisingly varied across different genera. For example, *Penicillium* species tend to reduce postprandial glucose elevations by inhibiting α-glucosidase and α-amylase [71], *Colletotrichum gloeosporioides* has been reported to enhance insulin secretion and improve glucose uptake in muscle cells [72], and metabolites from *Pestalotiopsis* strains show improvements in glucose tolerance and insulin sensitivity in experimental models [73]. Other endophytes, such as those associated with Dendrobium officinale, appear to modulate insulin-signaling pathways directly, potentially enhancing cellular glucose utilization [74]. These findings suggest that the metabolic profile of endophytes is far broader and more layered than typically expected from a single microbial group [14]. The reported antidiabetic and metabolic effects are supported by varying levels of experimental evidence, ranging from studies using crude fungal extracts to investigations of purified metabolites. Consequently, the interpretation of the underlying molecular mechanisms should consider the nature of the tested material, as biological activities observed with crude extracts may not always be attributable to a single bioactive constituent. Nevertheless, the available evidence collectively suggests that endophyte-derived metabolites have the potential to modulate multiple pathways involved in glucose homeostasis, insulin sensitivity, oxidative stress, and metabolic regulation [75].

Several endophyte-derived metabolites contribute to glycemic regulation through enzyme-level inhibition, particularly by targeting carbohydrate-digesting enzymes such as α-glucosidase and α-amylase, thereby reducing postprandial glucose excursions [76]. Enzyme-level inhibition of α-glucosidase and α-amylase has been directly modulated to carbohydrate processing in the gut, softening the glycemic impact of meals and reducing the metabolic load on the liver [77]. Others influence deeper metabolic regulators, including PPARγ and AMPK, which play central roles in insulin sensitivity and cellular energy balance [78]. Several fungal metabolites also seem to counteract oxidative stress through Nrf2-linked antioxidant mechanisms, a pathway known to protect pancreatic β-cells and maintain glucose homeostasis [79]. Interference with AGE–RAGE signaling, which is a major driver of diabetic vascular and hepatic complications, is another recurring mechanism reported. Studies also reveal that several endophyte-derived molecules appear to inhibit glycation or the downstream oxidative cascades associated with AGE accumulation [14]. Collectively, these complementary mechanisms suggest that endophyte-derived metabolites exert metabolic benefits through coordinated regulation of digestive enzymes, antioxidant defenses, insulin signaling, and glycation pathways, supporting their potential as multi-target therapeutic agents for metabolic disorders.

These observations together indicate that the metabolic influence of endophyte metabolites extends well beyond classical enzyme inhibition. Their collective involvement in AMPK, PPARγ, Nrf2, and AGE-RAGE pathways suggests that fungal metabolites may shape glucose regulation in a more systemic and coordinated fashion than previously appreciated. Although many mechanistic details remain incomplete, the emerging pattern points to a layered combination of antioxidant, anti-inflammatory, and insulin-sensitizing effects that could be relevant for chronic metabolic disorders.

### 3.3. Antioxidant-Linked Anti-Inflammatory Activity

Endophytic organisms are reported as important sources of natural chemical structures that possess antioxidant activity [80]. Studies across terrestrial, marine, and mangrove habitats show notable antioxidant activity, which includes *Chaetomium globosum*, *Trichoderma citrinoviride*, various *Penicillium* and *Aspergillus* species, *Epicoccum nigrum*, *Alternaria alternata*, and *Fusarium solani* [18,81,82,83,84], identifying metabolites with strong radical-scavenging activity. These reports underscore the diverse fungal endophytes and their potential as promising natural sources of antioxidants. The relationship between antioxidants and inflammatory immune-related disorders has garnered considerable interest in recent years, as antioxidants play a vital role in eliminating these oxidative byproducts [85]. In normal cellular conditions, reactive oxygen species (ROS) and reactive nitrogen species (RNS) are highly reactive molecules; for instance, ROS and RNS have the potential to interfere with mitochondrial respiration, leading to damage of essential biological macromolecules like proteins and DNA [86].

Inflammation is fundamentally a protective response; however, it can easily shift into something more damaging. Reports revealed that prolonged or dysregulated inflammation has been linked to chronic illnesses ranging from cardiovascular disease to autoimmune disorders and certain cancers [87]. Keeping this response in check is therefore essential for maintaining overall health. Endophyte-derived metabolites may contribute to this balance, not only by reducing inflammatory signals but also by shaping how cells interpret stress. Several studies reported that many endophytic compounds appear to lower the production of key pro-inflammatory mediators, supporting a more measured immune response that helps sustain homeostasis [88]. Polyphenols, terpenoids, and related metabolites have repeatedly shown an ability to modulate inflammatory pathways, and their presence in endophytic fungi suggests a broader biological role than previously assumed [89]. The reported antioxidant and anti-inflammatory activities are supported by studies employing both crude fungal extracts and purified metabolites. While crude extracts frequently demonstrate reductions in oxidative stress markers and inflammatory mediators, mechanistic investigations using purified compounds provide stronger evidence for the modulation of specific signaling pathways. Therefore, the reported biological activities should be interpreted in the context of the experimental model, the level of metabolite purification, and the degree of mechanistic validation [90].

Studies revealed that Lasiodiplactone A from *Lasiodiplodia theobromae* suppresses nitric oxide in LPS-stimulated macrophages (IC_50_ ≈ 23.5 µM) and also inhibits α-glucosidase (IC_50_ ≈ 29.4 µM), hinting at a small but meaningful intersection between inflammatory control and metabolic regulation [91]. Mazumder and his colleagues [92], reported that several endophytes residing *Elaeocarpus floribundus*, including *Aspergillus fumigatus*, *A. niger*, *Rhizoctonia oryzae*, *Rhizopus oryzae*, and *Syncephalastrum racemosum*, produced extracts with analgesic and anti-inflammatory activity. A similar observation was made by [88], who found notable anti-inflammatory activity in *Penicillium brefeldianum* isolated from *Acalypha hispida*. Other metabolites like Botryosphaerin B were reported from *Botryosphaeria* sp. inhibits COX-2, and Cyclonerodial B from *Trichoderma* sp. reduces nitric oxide in microglia, raising the possibility of neuroprotective applications [55]. Extracts of *Cytospora rhizophorae* isolates further lower NO, IL-6, and TNF-α, pointing to wider cytokine-level regulation. *Aspergillus niger*, *Rhizopus oryzae*, *Dendryphion nanum*, *Pleospora tarda*, and various *Penicillium* species have also shown inhibition of COX-1, COX-2, and 5-lipoxygenase, and some produce herbarin, a compound already recognized for its anti-inflammatory effects [93,94].

### 3.4. Immunomodulatory Effects

**Figure 2:**
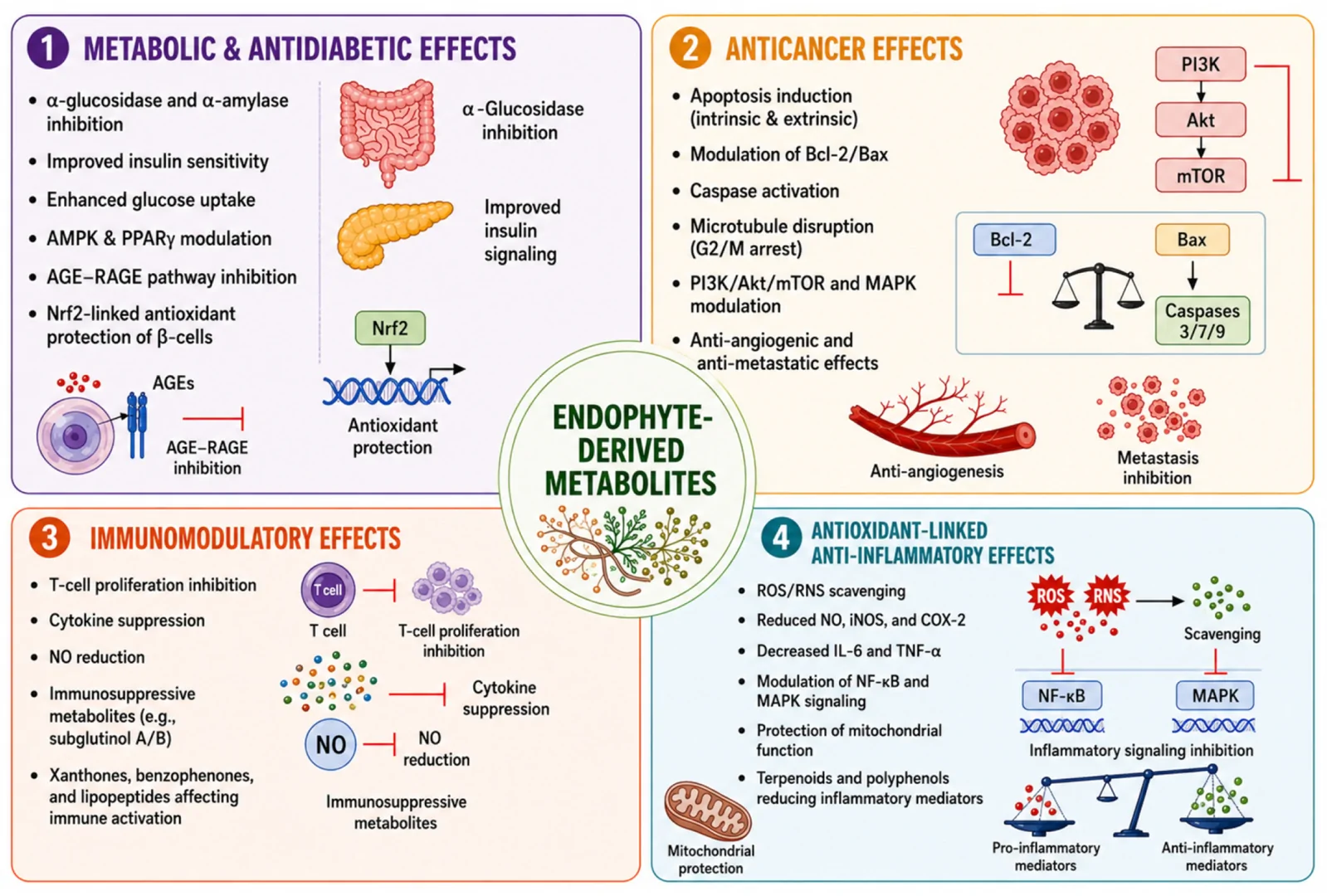
Pathway-level biological activities of endophyte-derived metabolites in chronic diseases and aging. This schematic represents a conceptual synthesis based predominantly on in vitro studies and selected in vivo models and is intended to illustrate pathway-level convergence rather than quantitative or pharmacokinetic comparisons (Created in BioRender. Nazir, A. (2026) https://BioRender.com/o22a6q4).

Several reports suggest that fungal endophytes might also serve as a reservoir of potential immunosuppressive agents, an area where safer alternatives are urgently needed. Compounds such as colutellin A, dibenzofuran derivatives, lipopeptides, sydoxanthones, and subglutinol A and B have demonstrated immune-modulating activity in early studies [53]. Endophytes from *Psidium guajava* and *Newbouldia laevis* produce citrinin, nidulalin, *p*-hydroxybenzoic acid, and cyclopenin, each associated with suppressed immune activation [95]. More targeted effects have been observed with mycousnine enamine from *Mycosphaerella nawae*, which inhibits T-cell proliferation by reducing CD25 and CD69 expression. *Penicillium* sp. ZJ-SY2 were also reported to produce benzophenones and xanthones with strong immunosuppressive activity [96]. Subglutinol A/B from *Fusarium subglutinans* and Albifpyrrols B from *Albifimbria viridis* also contribute to this emerging profile, the latter reducing LPS-driven B-cell proliferation [97]. Libertellenone J from *Phomopsis* sp. S12 suppresses NO and multiple cytokines, while extracts of *Botryosphaeria dothidea* BAK-1 show dose-dependent inhibition of T-cell responses. Although early-stage, these findings collectively suggest that endophytes may offer a structurally diverse pool of immunosuppressive molecules with therapeutic potential [58]. It is important to note that the immunomodulatory activities described are supported by studies employing different experimental systems, including crude fungal extracts, isolated metabolites, and purified compounds. While several purified metabolites have demonstrated specific effects on immune cell signaling and cytokine production (Figure 2), many observations remain based on preliminary in vitro studies [53].

### 3.5. Antimicrobial Potential and Relevance to Antibiotic Discovery

Endophytic fungi have emerged as an important source of structurally diverse metabolites with demonstrated antimicrobial activity against a broad range of bacterial and fungal pathogens. Beyond demonstrating broad-spectrum antimicrobial activity, several endophyte-derived metabolites have been reported to exert their effects through distinct molecular mechanisms. These include disruption of microbial cell membrane integrity, inhibition of cell wall and nucleic acid synthesis, interference with protein biosynthesis, induction of oxidative stress, and attenuation of quorum sensing and biofilm formation. Such diverse mechanisms may contribute to activity against multidrug-resistant pathogens and reduce the likelihood of resistance development compared with agents acting through a single molecular target. However, for many metabolites, these mechanisms remain incompletely characterized and require further validation using purified compounds and mechanistic studies [27,53]. In addition to their anticancer, antioxidant, and anti-inflammatory properties, many endophyte-derived compounds exhibit inhibitory effects against clinically relevant microorganisms, highlighting their potential as a source of novel anti-infective agents. Given the growing global challenge of antimicrobial resistance (AMR), there is increasing interest in exploring microbial natural products from underutilized ecological niches, including endophytic fungi, for antibiotic discovery.

Numerous endophytic fungal genera, including *Aspergillus*, *Penicillium*, *Fusarium*, *Chaetomium*, *Trichoderma*, and *Pestalotiopsis*, have been reported to produce metabolites with antibacterial and antifungal activities [27,53,98]. These metabolites belong to diverse chemical classes such as alkaloids, terpenoids, polyketides, phenolics, and peptide-derived compounds, many of which possess unique structural scaffolds not commonly observed in conventional antibiotics. Such chemical diversity provides opportunities for identifying new mechanisms of antimicrobial action and overcoming existing resistance pathways.

Several studies have demonstrated the activity of endophyte-derived metabolites against important human pathogens, including *Staphylococcus aureus*, *Escherichia coli*, *Pseudomonas aeruginosa*, and *Candida albicans* [32,53,81]. In addition to direct antimicrobial effects, some metabolites exhibit antibiofilm activity and interfere with microbial virulence mechanisms, thereby enhancing their therapeutic potential. The antimicrobial properties of endophytes may also contribute to the ecological fitness of their host plants by protecting them against invading pathogens, suggesting that these metabolites have evolved as natural defense molecules with potential translational applications in human medicine.

Interestingly, several signaling pathways discussed throughout this review, including NF-κB, MAPK, and oxidative stress-associated pathways, are also involved in host responses to microbial infections. Therefore, endophyte-derived metabolites may offer dual benefits by exerting direct antimicrobial activity while simultaneously modulating host inflammatory and immune responses. This combined antimicrobial and immunomodulatory profile may be particularly valuable in the management of infection-associated inflammatory disorders.

The integration of metabolomics, genome mining, molecular networking, and artificial intelligence-based screening approaches is expected to accelerate the discovery of novel antimicrobial metabolites from endophytic fungi. Representative examples of endophytic fungi, their bioactive metabolites, and reported antimicrobial activities are summarized in Table 2. Collectively, these findings highlight the potential of endophyte-derived metabolites as a valuable source of novel antimicrobial agents with broader therapeutic applications. As resistance to existing antibiotics continues to increase worldwide, endophytes represent a promising and relatively underexplored reservoir of bioactive compounds that could contribute to the development of next-generation antimicrobial therapeutics. Beyond their direct antimicrobial effects, many endophyte-derived metabolites have also been reported to influence microbial communities and host–microbe interactions. This emerging connection between endophyte metabolites and microbiome modulation has attracted growing interest, particularly because microbial homeostasis plays a central role in immune regulation, metabolic health, and chronic disease prevention.

**Figure 3:**
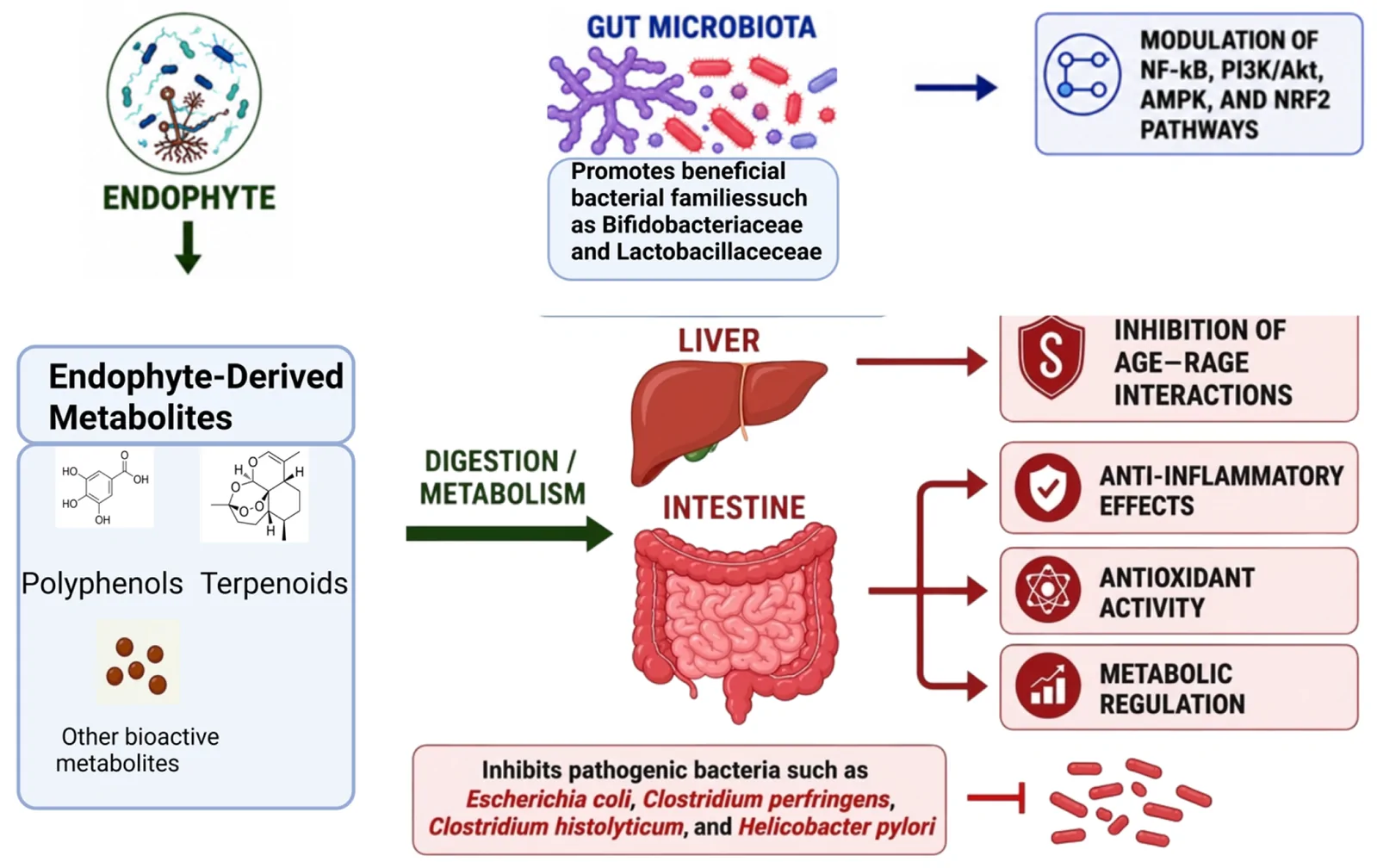
Interactions between endophyte-derived metabolites, gut microbiota, and host metabolic-inflammatory pathways (Created in BioRender. Nazir, A. (2026) https://BioRender.com/6ern7xu).

Polyphenols, flavonoids, and terpenoids are increasingly recognized for the way they shape gut ecology (Figure 3). Studies have demonstrated that polyphenols can influence intestinal ecology and maintain gut microbial balance by exerting: (i) a prebiotic effect that promotes the growth and establishment of probiotic bacterial families like Bifidobacteriaceae and *Lactobacillaceae*, and (ii) an antimicrobial activity that inhibits pathogenic bacteria such as *Escherichia coli*, *Clostridium perfringens*, *Clostridium histolyticum*, and *Helicobacter pylori*, a pattern that appears central to their gut-protective effects [100]. After ingestion, only a fraction of these compounds is absorbed in the upper gut, while the remainder reaches the colon, where microbes perform transformations such as hydrolysis and reduction [101]. These microbial conversions often expand biological activity, generating bioactive metabolites that can act locally or systemically [102].

These metabolites can strengthen gut barrier function, enhance mucosal immunity, and modulate host metabolism by altering microbial balance [103], positions them as important contributors in the context of conditions such as inflammatory bowel disease, obesity, diabetes, and cardiometabolic dysfunction [104]. Emerging work also suggests consequences for the gut–brain axis, reflecting the wider systemic reach of microbiota-derived metabolites.

An individual’s microbial profile, the dose and bioavailability of the compound, and the specific mechanisms involved, whether antioxidant activity, suppression of pro-inflammatory pathways, modulation of NF-κB, or interference with AGE-age-related signaling, play an important role [105]. Supplementation with polyphenols has also been reported to have a lower risk of gastrointestinal disorders, such as irritable bowel syndrome and infection-related diarrhea [106]. Overall, these findings indicate that the sequence of events, including digestion, microbial transformation, changes in microbiota, and host response, constitutes a multi-step pathway through which phytochemicals provide their gut-related benefits.

Endophyte-derived metabolites, following digestion and metabolism, may interact with the gut microbiota and host tissues, promoting beneficial bacterial families while suppressing pathogenic species. These interactions are associated with modulation of key signaling pathways, including NF-κB, PI3K/Akt, AMPK, and Nrf2, inhibition of AGE–RAGE interactions, and downstream antioxidant, anti-inflammatory, and metabolic regulatory effects in intestinal and hepatic systems. This figure represents a conceptual framework based predominantly on in vitro studies and selected in vivo evidence and is intended to illustrate pathway-level interactions rather than quantitative, pharmacokinetic, or exposure-based comparisons.

## 4. Endophyte Metabolites in Anti-Aging and Health Span

Reports suggest that aging will become a significant social issue globally in the upcoming decades [107]. As individuals age, bodily tissues and organs experience a decline in function, age-related illnesses and reducing their healthy lifespan become more vulnerable, thereby imposing significant financial burdens on nations globally regarding pensions, healthcare costs, and medical treatment [108,109]. Health span, defined as the period of life lived in good functional health without chronic disease, is now considered a more meaningful measure than lifespan in aging research. Therefore, investigating the biological aspects of aging, seeking safe and effective methods to positively influence health conditions, and extending the healthy lifespan of older individuals are crucial for alleviating the global pension strain and encouraging healthy aging. Beyond oxidative stress and chronic inflammation, aging is increasingly understood through interconnected biological hallmarks, including cellular senescence, impaired autophagy, mitochondrial dysfunction, and loss of proteostasis [110]. These processes reinforce one another and collectively contribute to functional decline, tissue degeneration, and increased susceptibility to chronic diseases. Consequently, therapeutic agents capable of simultaneously modulating multiple aging-associated pathways are receiving growing attention within geroscience research [108,109]. These hallmarks tend to reinforce one another, which may explain why interventions that influence redox balance or metabolic signaling often show benefits across multiple age-linked conditions. Chronic low-grade inflammation is another key driver of age-associated decline, fueled in part by persistent oxidative stress and metabolic dysfunction. Endophyte metabolites that dampen inflammatory signaling, therefore, have the potential to influence multiple aging hallmarks simultaneously. Cellular senescence is another hallmark of aging characterized by irreversible cell-cycle arrest and the accumulation of senescence-associated secretory phenotype (SASP) factors that promote chronic inflammation and tissue dysfunction [110]. Although direct evidence remains limited, several endophyte-derived phenolics, flavonoids, and terpenoids have demonstrated antioxidant and anti-inflammatory properties that may indirectly attenuate senescence-associated pathways by reducing oxidative stress and suppressing pro-inflammatory cytokine production [108]. Further mechanistic studies are required to determine whether these metabolites directly regulate cellular senescence pathways. Notably, several endophyte-derived metabolites interface with major hallmarks of aging, including oxidative stress, chronic inflammation, proteostasis imbalance, mitochondrial dysfunction, and metabolic rigidity. Initiatives aimed at discovering medications that enhance health span by addressing the mechanisms of aging have increasingly gained attention in this domain [109]. Endophyte metabolites appear to touch many of these same pressure points. Their behavior occasionally resembles that of well-studied gut microbial metabolites such as short-chain fatty acids, indole derivatives, or urolithins, especially in the way they interact with antioxidant defense systems or modulate inflammatory tone [111]. Increasing evidence indicates that Reactive Oxygen Species (ROS), for example, O_2_•^−^ and •OH and free radical-mediated reactions can cause oxidative damage to biomolecules (for example, lipids, proteins, and DNA), eventually contributing to aging, cancer, atherosclerosis, and other neurodegenerative disorders [112].

**Figure 4:**
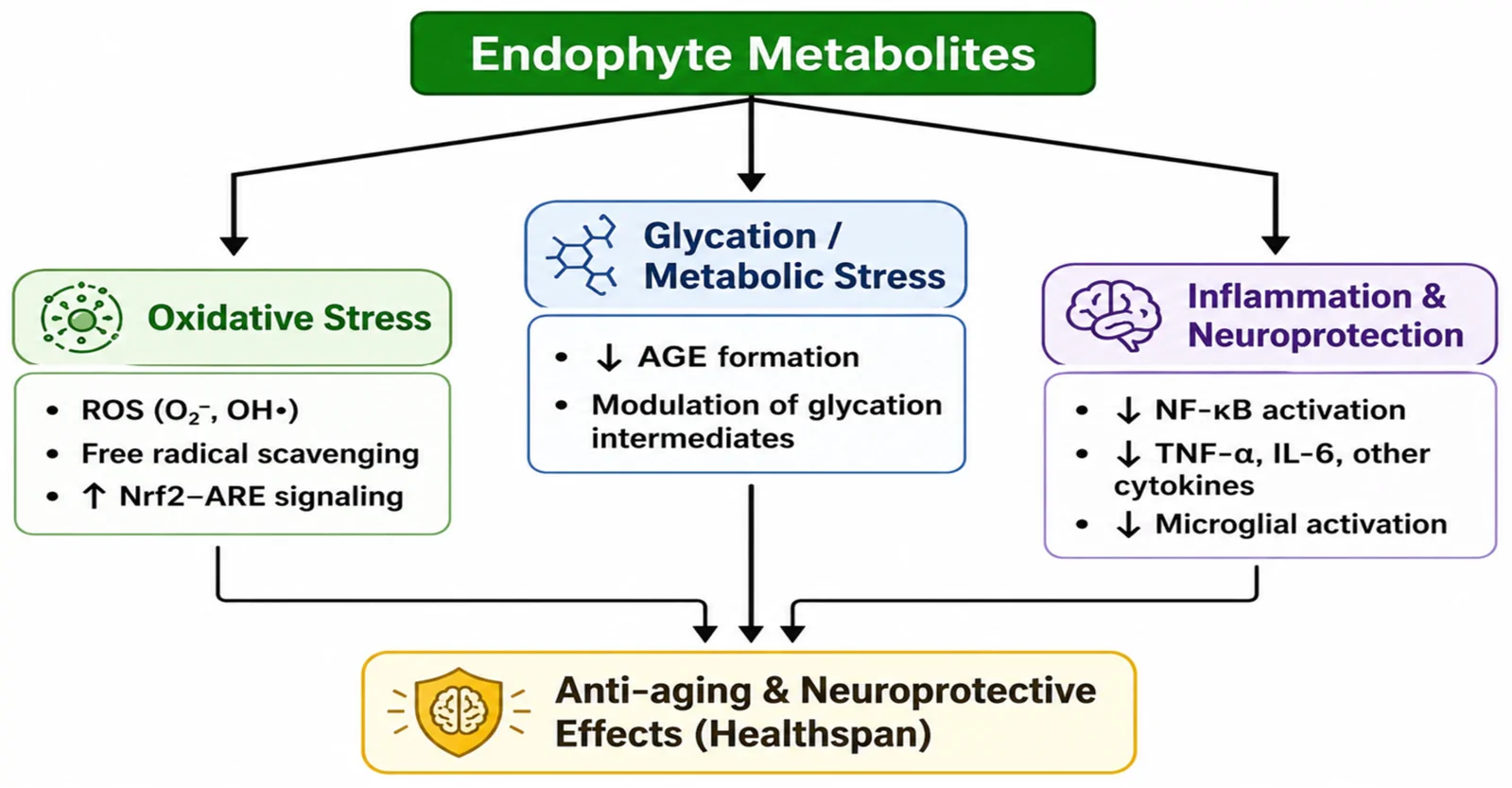
Role of endophyte-derived metabolites in oxidative stress regulation, glycation control, and inflammation-linked neuroprotection (Created in BioRender. Nazir, A. (2026) https://BioRender.com/5q14klr).

Studies show that endophyte-derived phenolics and flavonoids also continue to attract attention because they consistently show strong radical-scavenging activity, and this activity appears increasingly relevant in the context of aging biology. As several studies suggest, these metabolites may contribute to redox balance not only by neutralizing free radicals but also by shaping cellular responses linked to stress adaptation [57]. Phenolic-rich extracts from *Penicillium*, *Aspergillus*, and *Fusarium* endophytes have demonstrated potent antioxidant capacity, highlighting their direct contribution to oxidative stress resistance [18,113]. Reports also show that reactive oxygen species (ROS) are produced continuously even under normal physiological conditions, and while they play a role in host defense, excessive accumulation can damage lipids, proteins, and nucleic acids, eventually driving cancers, neurodegenerative disorders, diabetes, and broader features of aging [114,115]. Reports of endophyte-derived antioxidants often describe both primary antioxidant mechanisms, direct hydrogen or electron transfer, and secondary mechanisms such as metal chelation or interference with redox cycling [56], suggesting that their actions may be more layered than initially assumed. Reports also show that some metabolites also appear to activate Nrf2-dependent signaling, which supports long-term antioxidant resilience rather than short-term radical quenching [54]. As illustrated in Figure 4, endophyte-derived metabolites influence multiple aging-related pathways. Endophyte-derived metabolites influence key processes associated with aging and health span by reducing oxidative stress, modulating glycation and metabolic stress, and suppressing inflammatory signaling. These coordinated effects converge on antioxidant defense, attenuation of AGE formation, and reduced activation of pro-inflammatory mediators, collectively contributing to anti-aging and neuroprotective outcomes. Several endophyte-derived compounds have also been reported to modulate NF-κB–dependent pathways and reduce the production of pro-inflammatory cytokines, suggesting a direct impact on systemic and tissue-level inflammatory tone [116]. There is a link between oxidative stress and glycation that provides an additional angle, through which these compounds may influence aging. Protein glycation, and the subsequent formation of advanced glycation end products (AGEs), is closely associated with diabetic complications and with several hepatic and cardiovascular features of aging [117]. Because AGEs can intensify vascular inflammation, microglial activation, and neuronal damage, antiglycation activity may translate into both metabolic and neuroprotective benefits. Phenolic and flavonoid metabolites from endophytes frequently inhibit AGE formation, an effect attributed partially to their antioxidant behavior and partly to more specific interactions with intermediate glycation pathways [50]. Several isolates, including those producing phenolic-rich extracts, have demonstrated both ROS-scavenging and antiglycation effects [118,119,120]. Because AGEs amplify inflammation, oxidative damage, and metabolic dysfunction, inhibiting their formation provides a direct pathway for influencing health span and delaying age-associated decline. These dual activities, maintaining redox homeostasis and limiting protein glycation, position endophyte metabolites as promising contributors to antioxidant defenses, metabolic stability, and liver-related aging processes. Maintenance of proteostasis and efficient autophagy is equally important for healthy aging, as these processes facilitate the removal of damaged proteins and dysfunctional organelles while preserving cellular homeostasis [110]. Emerging evidence suggests that several natural products, including endophyte-derived metabolites, may influence autophagy-related signaling through AMPK and mTOR pathways while simultaneously reducing oxidative stress and chronic inflammation. However, direct evidence linking individual endophyte-derived metabolites with regulation of autophagy and proteostasis remains limited, highlighting an important area for future investigation. Overall, the ability of endophyte metabolites to act on multiple aging mechanisms simultaneously highlights their potential as multi-target natural agents for supporting healthy aging and extending functional lifespan. By simultaneously buffering oxidative stress, restraining inflammatory cascades, and protecting neurons from glyco-oxidative damage, these metabolites emerge as candidates for integrated anti-aging and neuroprotective strategies.

## 5. Challenges and Limitations

Despite the promise of endophyte-derived metabolites, several limitations still hinder their progress toward pharmacological use. Instability in metabolite production always remains an ongoing issue. The same fungal strain can produce different chemical profiles even with small changes in the culture environment, such as media composition, aeration, or stress conditions [121,122]. This variability makes it difficult to reproduce results across labs. Additionally, silent or weakly expressed biosynthetic gene clusters pose a challenge, as many metabolites go undetected unless specific triggers or co-culture strategies are employed [16].

Much of the current literature relies heavily on in vitro assays, including those for antioxidants, cytotoxicity, or enzyme inhibition, often using crude extracts [92,123]. While these screens are useful for initial discovery, they do not always capture mechanistic insight, bioavailability, or dose relevance. Toxicity also remains insufficiently characterized. Although several endophyte-derived compounds, including quinazolinones, diterpenoids, and diketopiperazines, have demonstrated cytotoxic or immunosuppressive activities that may limit their therapeutic application [24,58,124], systematic evaluations of acute toxicity, chronic toxicity, pharmacokinetics, and long-term safety in animal models remain scarce. Addressing these knowledge gaps will be essential before endophyte-derived metabolites can progress toward clinical translation.

Scalability and regulation add further barriers. Fermentation yields often fluctuate, and downstream purification is not trivial, especially when metabolites occur in trace quantities [19]. From a regulatory perspective, endophyte metabolites sit at the intersection of natural products, biologics, and microbiome-derived therapeutics, and guidelines for standardization remain unclear [10].

The final limitation is conceptual. Many studies analyse metabolites in isolation, even though multiple fungal compounds may interact or require host–microbe context to reveal their full activity [7]. Advanced models, co-culture systems, plant-based media [29], or metabolomics-guided workflows [125] are still underused, leaving important ecological and pharmacological interactions unexplored. In addition, greater standardization of metabolite isolation, purification, structural characterization, and biological validation is required to improve reproducibility across studies. Harmonized experimental protocols and the integration of metabolomics, transcriptomics, and functional assays will facilitate more reliable comparisons between studies and accelerate the translation of promising endophyte-derived metabolites into clinically relevant therapeutics.

## 6. Future Perspectives

More integrated experimental approaches could help move the field forward. Genome mining and metabolomics offer a promising route for linking biosynthetic gene clusters to their chemical products, and tools such as LC–MS/MS molecular networking are already helping to map families of related metabolites before purification begins. Activating silent biosynthetic gene clusters through epigenetic modifiers or CRISPR/Cas-based genome editing represents a promising strategy for uncovering novel bioactive metabolites that remain inaccessible under conventional culture conditions. Coupled with genome mining and metabolomic profiling, these approaches can accelerate the discovery, functional characterization, and optimization of endophyte-derived therapeutic compounds.

Co-culture strategies deserve particular attention. Several studies show that endophytes alter their metabolic output when grown alongside other fungi or when exposed to mild stress, a pattern seen in both terrestrial and mangrove-associated isolates. Using host-derived substrates, which more closely mimic native conditions, may also improve metabolite diversity and relevance. Co-cultivation with competing microorganisms or host-associated microbes has emerged as an effective strategy for activating cryptic biosynthetic gene clusters that remain silent under conventional laboratory conditions. This approach enhances metabolite diversity by stimulating interspecies interactions and stress-responsive secondary metabolism, thereby increasing the likelihood of discovering novel bioactive compounds with therapeutic potential [126].

Future pharmacological validation should expand beyond 2D cell lines. More physiologically relevant models, organoids, immune–epithelial co-culture systems, or animal models could help clarify how endophyte metabolites influence inflammation, oxidative pathways, or metabolic signaling under conditions closer to human physiology. In aging research, models that capture redox instability, mitochondrial shifts, or chronic inflammatory tone will be especially valuable.

There is also room to integrate endophyte-focused work with microbiome-driven therapeutics. Several fungal metabolites resemble gut microbial products in the way they shape redox balance, immune tone, and metabolic regulation. Studying these systems together may reveal shared therapeutic circuits and point toward hybrid interventions that combine plant-associated and host-derived microbial metabolites.

Lastly, advancing this field will require stronger collaboration between natural-product chemists, microbiologists, pharmacologists, and systems biologists. As new tools emerge including metabolomics, genome mining, co-culture engineering, and synthetic biology the opportunities to identify, validate, and eventually translate endophyte-derived metabolites into therapeutic candidates are likely to expand significantly.

### Emerging Technologies in Endophyte-Based Drug Discovery

Emerging technologies including metabolomics, genome mining, and artificial intelligence (AI)-assisted drug discovery are expected to accelerate the identification and characterization of endophyte-derived metabolites [127]. Metabolomics-based profiling and LC–MS/MS molecular networking enable rapid detection of structurally related compounds, while genome mining approaches facilitate the identification of silent biosynthetic gene clusters associated with novel metabolite production. In parallel, AI-assisted screening, predictive modeling, and systems pharmacology approaches may help prioritize bioactive compounds, predict pathway interactions, and support the development of multi-target therapeutics for chronic diseases and healthy aging [128].

## 7. Conclusions

Endophytic fungi represent a promising and still underexplored source of bioactive metabolites with the potential to modulate multiple pathways involved in chronic diseases and aging. The diverse chemical classes produced by these organisms, including alkaloids, terpenoids, polyketides, and phenolic compounds, have shown the ability to influence key signaling pathways such as NF-κB, Nrf2, PI3K/Akt, AMPK, and AGE–RAGE. This multi-target activity highlights their potential as candidates for addressing complex, pathway-driven diseases.

At the same time, several limitations need to be considered. Variability in metabolite production, limited reproducibility across studies, and the lack of detailed pharmacokinetic and toxicity data remain important challenges. Much of the current evidence is still based on in vitro studies, and further validation in physiologically relevant models is required. Beyond their roles in chronic disease modulation and healthy aging, endophyte-derived metabolites also represent a promising source of antimicrobial compounds with potential applications in antibiotic discovery. Their structural diversity, together with demonstrated antimicrobial, immunomodulatory, and microbiome-associated activities, highlights their value as candidates for the development of next-generation anti-infective and multi-target therapeutic strategies. Continued exploration of these metabolites may contribute to addressing emerging challenges associated with antimicrobial resistance while expanding the repertoire of naturally derived therapeutics.

Advances in metabolomics, genome mining, and co-culture approaches offer new opportunities to better understand and expand the chemical and functional diversity of endophyte-derived metabolites. Integrating these tools with more advanced biological models may help bridge the gap between experimental findings and clinical relevance.

Overall, while significant challenges remain, endophyte-derived metabolites represent a valuable and evolving platform for the development of multi-target therapeutic strategies targeting chronic diseases and promoting healthy aging. Their structural diversity and ability to modulate interconnected pathways associated with oxidative stress, inflammation, metabolic dysfunction, glycation, and antimicrobial resistance underscore their potential within emerging geroscience and systems pharmacology approaches. However, successful clinical translation will require standardized metabolite characterization, rigorous mechanistic validation, comprehensive safety evaluation, and well-designed in vivo and clinical studies. Future integration of artificial intelligence-assisted discovery, metabolomics, genome mining, synthetic biology, and CRISPR/Cas-based genome editing is expected to accelerate the discovery, optimization, and clinical development of endophyte-derived therapeutics.

## Figures and Tables

**Table 2:** Representative Endophyte-Derived Antimicrobial Metabolites, Their Target Pathogens, Proposed Mechanisms, and Additional Pharmacological Activities.

Endophytic Fungus	Representative Metabolite(s)	Target Pathogen(s)	Proposed Antimicrobial Mechanism	Additional Pharmacological Activity	Reference
*Aspergillus fumigatus*	Phenolic compounds, flavonoid-like metabolites	*Staphylococcus aureus*, *Escherichia coli*, *Candida albicans*	Reported inhibition of microbial growth associated with antioxidant activity	Antioxidant, anti-inflammatory	[39,81]
*Penicillium* spp.	Alkaloids, terpenoids, diketopiperazines	Gram-positive and Gram-negative bacteria	Proposed membrane disruption and inhibition of microbial growth	Immunomodulatory	[82,98]
*Fusarium* spp.	Chaetominine, phenolics, polyketides	*Staphylococcus aureus*, *Bacillus subtilis*	Reported inhibition of bacterial proliferation	Anticancer, immunomodulatory	[53]
*Chaetomium* spp.	Chaetominine and related alkaloids	*Staphylococcus aureus*, *Candida albicans*	Reported inhibition of microbial growth	NF-κB modulation, anticancer	[53]
*Trichoderma* spp.	Diketopiperazines, Cyclonerodial B	*Candida albicans*, filamentous fungi	Proposed disruption of fungal cell integrity	Anti-inflammatory, cytokine modulation	[55]
*Pestalotiopsis* spp.	Secondary metabolites	*Escherichia coli*, *Pseudomonas aeruginosa*, *Staphylococcus aureus*	Broad-spectrum inhibition of microbial growth	Antidiabetic, metabolic regulation	[73]
*Botryosphaeria* spp.	Botryosphaerin B	Gram-positive bacteria	Reported antibacterial activity; precise mechanism remains unclear	COX-2 inhibition, anti-inflammatory	[55]
*Lasiodiplodia theobromae*	Lasiodiplactone A	*Staphylococcus aureus*, *Escherichia coli*	Reported inhibition of microbial growth	α-Glucosidase inhibition, anti-inflammatory	[99]

## Data Availability

No new data were created or analyzed in this study. Data sharing is not applicable to this article.
